# Infertility Prevention and Health Promotion: The Role of Nurses in Public Health Initiatives

**DOI:** 10.31661/gmj.v13i.3534

**Published:** 2024-10-17

**Authors:** Malihe Sagheb Ray Shirazi, Fatemeh Salarkarimi, Fatemeh Moghadasi, Fatemeh Mahmoudikohani, Farnoosh Tajik, Zahra Bastani Nejad

**Affiliations:** ^1^ Department of Anatomical Sciences, Faculty of Nursing and Midwifery, Hormozgan University of Medical Sciences, Bandar Abbas, Iran; ^2^ Department of Midwifery, Reproductive Health Promotion Research Center, Ahvaz Jundishapur University of Medical Sciences, Ahvaz, Iran; ^3^ Department of Nursing, Arak School of Nursing, Arak University of Medical Sciences, Arak, Iran; ^4^ Department of Midwifery, School of Nursing and Midwifery, Bam University of Medical Sciences, Bam, Iran; ^5^ Nursing Care Research Center, Semnan University of Medical Sciences, Semnan, Iran; ^6^ Department of Pediatric and Neonatal Nursing, School of Nursing and Midwifery, Semnan University of Medical Sciences, Semnan, Iran; ^7^ Department of Nursing, School of Nursing, Fasa University of Medical Sciences, Fasa, Iran

**Keywords:** Infertility, Nurses, Reproductive Health, Lifestyle Interventions, Healthcare Policy, Patient Education, Community Outreach

## Abstract

Infertility is a growing public health concern, affecting millions of individuals and couples worldwide. Despite advancements in medical treatments, prevention remains a critical strategy for reducing the burden of infertility. Nurses, as frontline healthcare providers, play a pivotal role in infertility prevention and health promotion, particularly through public health initiatives. This review aims to explore the diverse roles of nurses in infertility prevention and their contributions to public health strategies. A review of existing literature was conducted to examine the epidemiology of infertility, key risk factors, and the preventive measures that can be employed by nursing professionals. Emphasis is placed on the role of nurses in health education, screening, early detection, and community-based interventions, which are essential in reducing infertility rates. In addition, this review identifies barriers that impede effective nurse-led infertility prevention, such as disparities in access to care, cultural sensitivity challenges, and policy constraints. Evidence suggests that nurses are well-positioned to lead public health campaigns, conduct reproductive health counseling, and advocate for policy reforms to improve infertility prevention. The review concludes with recommendations for future research, suggesting enhanced nursing education and training, as well as the need for stronger integration of nurses into public health policy-making. This study underscores the critical role of nurses in promoting reproductive health and preventing infertility, advocating for their inclusion in comprehensive public health strategies aimed at addressing infertility on a global scale.

## Introduction

infertility is often defined as the inability to conceive after regular, unprotected intercourse for a year or more. It is known to affect approximately 8-12% of couples worldwide [1]. As such, it is a complex issue with solid repercussions in social, emotional, and economic fields. World Health Organization recognizes that infertility is a significant public health issue that severely impacts people and societies globally [2]. The morbidity that comes with infertility is one of the primary sources of psychological distress, stigma, and relationship stress; it also usually requires complex, costly interventions [3]. Preventing infertility and promoting reproductive health are critical in reducing the burden associated with infertility. Interventions that are effective in avoiding infertility lead to enhancements in public health globally in the general population by reducing the modifiable risk factors related to lifestyle, environmental exposures, and access to healthcare services [4]. Health promotion efforts are oriented toward educating people about fertility preservation, timely medical consultation, and how leading a healthy life can help [5].

Public health interventions for preventing infertility and promoting health are a focal point in the work of nurses. Indeed, their extensive training and contact with the patients place them uniquely to both implement and advocate for comprehensive reproductive health programs [6]. Education, community outreach, early screening, and infertility intervention are essential for the nurses’ role [7]. They are also a vital link with general healthcare and can offer continuity and support in care to all patients. Nurses, by being knowledgeable and experienced in their field, can significantly contribute to improved reproductive health outcomes and reduce the incidence of infertility [8].

The purpose of this review is to examine essential public health nursing interventions that play a crucial role in infertility prevention. It offers valuable insights for policymakers, healthcare providers, and researchers, helping to develop effective strategies to address and reduce infertility.

## Epidemiology

**Table:** Table1. Summarized Some of the Most Critical Risk Factors Contributing to Infertility

**Risk Factor**	**Description**	**Reference**
**Age**	Fertility declines with age, particularly for women after age 35, as the quantity and quality of eggs diminish.	[13]
**Sexually Transmitted Infections (STIs)**	Infections like chlamydia and gonorrhea can lead to pelvic inflammatory disease (PID), which causes damage to reproductive organs.	[14]
**Obesity**	Excess body weight disrupts hormonal balance and ovulation, increasing the risk of infertility in both men and women.	[15]
**Smoking**	Tobacco use is associated with decreased sperm quality in men and impaired ovarian function in women.	[16]
**Alcohol and Drug Use**	Excessive alcohol consumption and drug use negatively impact reproductive function and reduce fertility.	[17][18]
**Environmental Toxins**	Exposure to chemicals, pesticides, and pollutants (e.g., heavy metals, endocrine disruptors) can affect sperm and egg quality.	[19][20]
**Poor Nutrition**	Deficiencies in critical nutrients (e.g., folate, zinc) can impair reproductive health and contribute to infertility.	[21]
**Stress**	Chronic stress disrupts hormonal balance and may reduce the likelihood of conception by affecting ovulation and sperm production.	[22][23]
**Polycystic Ovary Syndrome (PCOS)**	A common hormonal disorder affecting women, leading to irregular ovulation and reduced fertility.	[24]
**Endometriosis**	A condition where tissue similar to the uterine lining grows outside the uterus, leading to inflammation and infertility.	[25]

Globally, the World Health Organization (WHO) estimates that approximately 17.5% of reproductive-aged couples are affected by infertility, which equates to about 1 in 6 adults worldwide [9]. Historically, infertility was believed to vary significantly by region and socioeconomic factors, with higher rates observed in low- and middle-income countries (LMICs). However, recent estimates suggest minimal variation across regions, with comparable rates between countries [10]. This indicates that infertility is not confined to specific populations but is a major global health challenge. Lifetime prevalence is now reported at 17.8% in high-income countries and 16.5% in LMICs, reflecting the widespread nature of this condition [11].

While infertility rates are generally declining in high-income and developed countries, they appear to be increasing in other regions. This trend may be partly due to underreporting in developed countries, where reproductive intentions are lower, as well as better access to infertility treatments [12].

Since, Infertility is driven by a complex interaction of factors, including biological, environmental, and lifestyle influences (see Table-1). In recent decades, efforts to prevent infertility have increasingly focused on addressing modifiable risk factors, improving access to reproductive health services, and advancing public health education [26][27].

Public health campaigns have aimed to raise awareness about the importance of reproductive health, targeting behaviors such as smoking cessation, promoting healthy diets, and preventing Sexually Transmitted Infections (STIs) [28][29][30].

Countries with strong healthcare infrastructures, such as those in Europe and North America, have introduced preconception care programs, which encourage individuals to adopt healthier behaviors before trying to conceive [31][32]. These programs typically focus on lifestyle modification, screening for risk factors, and vaccinations against infections such as rubella and human papillomavirus (HPV), both of which are linked to infertility [32].

In LMICs, prevention efforts have been more focused on improving access to STI prevention and treatment [33]. These regions are particularly affected by infertility due to reproductive tract infections and inadequate healthcare access [34].Vaccination programs, community health education, and the promotion of family planning services are essential components of these efforts [35][36]. Additionally, policy changes advocating for workplace safety and reducing environmental toxin exposure have begun to emerge, especially in industrialized countries where environmental pollution and chemical exposures pose a significant threat to reproductive health [19][37].

## Nurses’ Role in Infertility Prevention and Health Promotion

**Table:** Table2. Major Public Health Nursing Interventions for Infertility Prevention

**Intervention**	**Objective**	**Methods**	**Expected Outcomes**
**Awareness**	Increase public knowledge about infertility risk factors and prevention strategies.	Conduct workshops, distribute educational materials, and use media to disseminate information on fertility risks (e.g., lifestyle, STIs).	Improved community awareness, resulting in healthier reproductive behaviors and reduced infertility risks.
**STI Screening and Prevention**	Reduce infertility caused by untreated STI.	Implement community-based STI screening programs, provide education on safe sexual practices, and offer early treatment options.	Lower rates of infertility due to early diagnosis and treatment of STIs such as chlamydia and gonorrhea.
**Preconception Counseling**	Promote healthy behaviors and lifestyle changes before conception to improve fertility outcomes.	Provide personalized counseling on weight management, nutrition, smoking cessation, and chronic disease management.	Increased adoption of preconception care practices, leading to improved fertility and pregnancy outcomes.
**Family Planning**	Assist individuals and couples in making informed decisions about reproduction and fertility.	Offer contraceptive counseling, fertility awareness education, and resources for managing family size and timing.	Improved reproductive health and family planning, reducing unintended pregnancies and infertility risks.
**Advocacy for Reproductive Health Policies**	Advocate for policies that improve access to reproductive health services, including infertility care.	Collaborate with policymakers to expand coverage for fertility treatments and screening services in public healthcare systems.	Enhanced access to affordable infertility prevention and treatment services, leading to lower rates of infertility.
**Community-Based Reproductive Health Programs**	Target at-risk populations with tailored reproductive health services and education.	Develop outreach programs, mobile health units, and culturally sensitive education to increase access to reproductive healthcare.	Increased use of reproductive health services in underserved areas, reducing infertility rates linked to lack of access.
**Environmental and Occupational Health Advocacy**	Reduce exposure to environmental and occupational hazards that contribute to infertility.	Conduct workplace assessments, advocate for stricter regulations on exposure to chemicals, and provide education on reproductive hazards.	Decreased infertility rates associated with environmental and occupational exposures, such as pesticides and toxins.

Nurses play a pivotal role in preventing infertility and promoting reproductive health, particularly through health education, screening, early detection, and community-based initiatives [8]. Their engagement in these areas can significantly reduce the burden of infertility by promoting awareness, facilitating early interventions, and advocating for accessible healthcare services.

Health Education

Nurses are at the forefront of educating individuals and communities about infertility risks and prevention strategies. By providing clear, accurate, and culturally sensitive information, nurses help individuals make informed decisions that can improve their reproductive health outcomes [38]. Key aspects of this role include:

1. Raising Awareness of Infertility Risk Factors

Nurses educate the public about the modifiable lifestyle factors that contribute to infertility, such as obesity, smoking, excessive alcohol consumption, and poor dietary habits [39]. They offer tailored advice on maintaining a healthy weight, quitting smoking, and adopting balanced diets rich in antioxidants to improve fertility outcomes [6][39]. Additionally, nurses emphasize the importance of avoiding environmental exposures that can affect fertility [40].

2. Promoting Safe Sexual Practices

Nurses provide education on safe sexual practices to reduce the incidence of STIs, a major contributor to infertility [41]. They encourage condom use and promote STI testing and treatment to prevent complications like pelvic inflammatory disease (PID) and tubal damage in women, and epididymitis in men [41][42][43].

3. Encouraging Preconception Health

Preconception health education is crucial for both men and women. Nurses promote preconception counseling that includes guidance on managing chronic conditions (e.g., diabetes, thyroid disorders) that can affect fertility [44]. They also stress the importance of timely family planning, particularly for women considering delayed childbearing, to mitigate the risks associated with advanced maternal age [13][38].

4. Addressing Cultural and Social Stigma

In many cultures, infertility is heavily stigmatized, which can prevent individuals from seeking help [45]. Nurses play a critical role in normalizing conversations around fertility, offering psychosocial support, and connecting patients to resources, thus reducing the psychological burden associated with infertility [46][47].

Screening and Early Detection

Early detection of infertility-related conditions is essential for timely intervention, and nurses are instrumental in leading these efforts through routine screening and assessments [48]. Some major points are below:

1. STI Screening

Nurse-led STI screening programs, particularly in vulnerable populations, are crucial in detecting and treating infections early to prevent long-term complications [49]. Regular STI testing in sexually active individuals can significantly reduce the risk of tubal blockages and other fertility issues linked to untreated infections [50].

2. Reproductive Health Screenings

Nurses conduct reproductive health assessments that include screening for conditions such as polycystic ovary syndrome (PCOS), endometriosis, and other gynecological disorders that can affect fertility [51][52]. Early identification of these conditions enables timely medical interventions that can improve the likelihood of conception [53].

3. Education on Fertility Awareness

Nurses also educate patients on fertility awareness methods (FAM), teaching individuals how to monitor ovulation cycles, understand fertility windows, and recognize potential signs of reproductive health issues [54].This empowers individuals to seek help early if they encounter irregularities in their menstrual cycles or difficulties in conceiving [38].

Community-based Initiatives

Nurses are uniquely positioned to design and implement community-based programs aimed at infertility prevention. These initiatives often target high-risk populations and focus on accessible, culturally relevant interventions [8][52]. There are some key strategies:

1. Outreach and Mobile Health Clinics

In resource-limited settings, nurses lead mobile health clinics that offer reproductive health services, including STI screening, fertility assessments, and education on reproductive health [43][55]. These mobile units are essential for reaching underserved populations who may not have easy access to healthcare facilities [56].

2. Group Education and Workshops

Nurses develop community workshops that provide group education on fertility preservation, contraception, and preconception health [57]. These workshops are often organized in partnership with local organizations, schools, and workplaces to reach a broad audience [58]. The group setting also allows for peer support and community discussion, which can reduce stigma and encourage collective action [59].

3. Culturally Tailored Programs

One of the major strengths of nurse-led community health programs is their ability to be culturally adapted [60]. Nurses work with community leaders and local stakeholders to design programs that are respectful of cultural beliefs and practices surrounding fertility [8].This ensures that educational content and health services are well-received and effective [61].

4. Collaborative Partnerships

Nurses often collaborate with public health agencies, non-governmental organizations (NGOs), and fertility clinics to implement comprehensive infertility prevention programs [62]. These partnerships allow for a multidisciplinary approach to addressing infertility by combining medical, educational, and psychosocial support services [52].

## Public Health Nursing Strategies for Infertility Prevention

Public health nurses play a crucial role in addressing infertility by implementing targeted strategies that promote reproductive health and prevent infertility [6]. These strategies encompass awareness campaigns, reproductive health counseling, and advocacy efforts, which collectively work to reduce infertility rates, particularly in high-risk populations [5][6]. Successful initiatives led by nurses demonstrate how these interventions can positively impact community health by raising awareness, facilitating early detection, and advocating for accessible reproductive healthcare [6][63]. Table-2 outlines the critical interventions led by public health nurses to prevent infertility.

Awareness Campaigns

One of the most effective strategies employed by public health nurses is the organization of awareness campaigns aimed at educating communities about infertility risks and prevention methods [64].

These campaigns typically focus on disseminating information about modifiable risk factors, such as lifestyle habits, sexual health, and environmental exposures [48][64].

For example, in communities with high rates of infertility due to untreated STIs, nurses lead campaigns that emphasize safe sexual practices, the importance of routine STI screening, and early treatment to prevent long-term reproductive damage [28][33][41].

Nurses also use awareness campaigns to address lifestyle-related factors, such as obesity, smoking, and alcohol consumption, all of which are known contributors to infertility [26][46].

By conducting community workshops, distributing educational materials, and utilizing media platforms, nurses raise awareness about the link between these factors and fertility, encouraging individuals to adopt healthier behaviors [57][58].

Reproductive Health Counseling

Reproductive health counseling provided by public health nurses is another critical strategy for infertility prevention [58]. This counseling is often delivered one-on-one or in small groups and covers a broad range of topics, including fertility awareness, family planning, and preconception care [44][58].

Nurses offer tailored advice on maintaining reproductive health, such as tracking ovulation cycles, optimizing preconception nutrition, and addressing chronic health conditions that may affect fertility, such as diabetes or thyroid disorders [32][44]. In many cases, nurses collaborate with local healthcare providers to offer integrated reproductive health services [55]. For example, in rural areas of sub-Saharan Africa, nurse-led initiatives have incorporated reproductive health counseling into broader maternal and child health services, ensuring that both men and women receive comprehensive education on fertility [65].

These programs have shown success in improving awareness of fertility-related issues and encouraging individuals to seek early medical intervention for reproductive health concerns [66].

Advocacy for Reproductive Health Policies

Advocacy is a key component of public health nursing strategies aimed at reducing infertility [67]. Nurses advocate for policy changes that ensure reproductive health services, including infertility prevention and treatment, are accessible and affordable for all [68]. This advocacy often focuses on improving access to care, particularly in underserved populations where financial and geographic barriers prevent individuals from receiving necessary infertility treatments [68][69].

A notable example of successful nursing advocacy is the federal STI prevention funding in the U.S., which was supported by public health nurses. This initiative focused on expanding STI screening and treatment programs, with an emphasis on chlamydia and gonorrhea, two leading causes of infertility [70]. The project’s success in reducing the prevalence of these infections in young women led to a marked decrease in cases of infertility associated with pelvic inflammatory disease (PID), demonstrating the impact of coordinated advocacy and prevention efforts [14][70].

## Challenges and Barriers in Infertility Prevention and Care

Infertility prevention and care face numerous challenges, with disparities in access, cultural sensitivity, and policy constraints hindering effective interventions. Nurses play a critical role in addressing these barriers by providing accessible care, promoting culturally sensitive education, and advocating for policy reforms that support reproductive health.

Access to Care

One of the most significant challenges in infertility prevention is unequal access to care, particularly in LMICs and underserved populations within high-income nations [71]. Financial barriers, geographical limitations, and inadequate healthcare infrastructure disproportionately affect access to infertility treatments and preventive care [72]. For many, assisted reproductive technologies (ART), such as in vitro fertilization (IVF), are prohibitively expensive and largely unavailable in public healthcare systems [73]. Additionally, limited access to reproductive health education and early screening services exacerbates the problem by delaying diagnosis and treatment of infertility-related conditions, such as STIs and gynecological disorders [71][74].

Nurses are essential in addressing these disparities through community outreach programs that offer education on reproductive health [75][76]. Mobile clinics and telemedicine initiatives, led by nurses, can bridge the gap for individuals in rural or remote areas [56]. Nurses also play a key role in advocating for universal healthcare policies that include infertility prevention and treatment as integral components of reproductive health services [77][78].

Cultural Sensitivity

Cultural beliefs and practices surrounding fertility are deeply embedded in many societies, particularly in regions where childbearing is closely tied to social status, gender roles, and identity [79]. In some cultures, infertility is associated with significant stigma, which can deter individuals from seeking care or discussing their reproductive health [45]. In patriarchal societies, women often bear the brunt of blame for infertility, even when male infertility is a contributing factor [80].

Nurses are uniquely positioned to provide culturally sensitive care by understanding and respecting these cultural dynamics [81]. To ensure that education and interventions are effective, nurses must tailor their communication strategies to reflect the beliefs, traditions, and language of the communities they serve [82]. This might involve collaborating with local community leaders, religious figures, and traditional healers to integrate reproductive health education into trusted community networks [8]. Gender-sensitive approaches are also vital, where nurses work to engage both men and women in conversations about fertility, recognizing that infertility is a shared issue and not solely the responsibility of women [80]. By reducing stigma and normalizing conversations around infertility, nurses can encourage early intervention and foster a more supportive environment for individuals facing reproductive challenges [83].

Policy Constraints

Policy-level barriers further complicate efforts to prevent and treat infertility [84]. In many countries, infertility care is not included in public health insurance, making access to fertility treatments and ART financially unattainable for a large portion of the population [74][85]. Additionally, the lack of standardized guidelines and protocols for infertility prevention and treatment contributes to inconsistencies in care [84]. Reproductive health policies often prioritize maternal and child health, while overlooking the need for comprehensive infertility services, leaving gaps in prevention strategies [84][86].

Nurses, as advocates for public health, can play a vital role in pushing for policy reforms that include infertility prevention and care in national and international healthcare agendas [78]. This includes advocating for the inclusion of fertility services in public health insurance schemes and promoting policies that fund public health education campaigns about infertility prevention[40][57]. Nurses can also support regulatory frameworks to limit exposure to environmental toxins linked to infertility, such as endocrine disruptors, and promote workplace policies that protect reproductive health [6][37].

Moreover, policy reforms should focus on ensuring gender equity in reproductive health services [87]. In many regions, fertility policies and services disproportionately focus on women, neglecting the critical need for male fertility screening and treatment [88]. Nurses can advocate for more balanced policies that address both male and female infertility, ensuring that interventions are holistic and inclusive [89].

Nurses are at the forefront of these efforts, offering culturally sensitive care, expanding access to reproductive health services, and advocating for policy reforms that prioritize infertility prevention and treatment [75]. By addressing these barriers, nurses can play a transformative role in reducing the global burden of infertility and improving reproductive health outcomes for all individuals.

## Future Directions and Recommendations

As infertility remains a significant public health challenge, there is a growing need for further research into the specific roles that nurses play in infertility prevention [10][48]. While existing interventions led by nurses have demonstrated positive outcomes, more comprehensive studies are required to better understand the full impact of nurse-led initiatives on reducing infertility rates [6][39]. Future research should explore the long-term effects of nurse-driven reproductive health programs, focusing on the efficacy of prevention strategies in diverse populations. Additionally, studies that examine the role of nurse-patient relationships in managing infertility-related stigma and psychosocial stress could provide valuable insights into improving patient-centered care.

To maximize the effectiveness of nurses in infertility prevention, improvements in nursing education and training are essential [41]. Nursing curricula should include specialized modules on reproductive health, with an emphasis on infertility risk factors, fertility awareness, and the psychosocial aspects of infertility [90]. Furthermore, training programs should prioritize practical skills, such as conducting STI screenings, delivering preconception counseling, and leading community health interventions [48][91]. Expanding the scope of nursing education to cover environmental health and occupational risks associated with infertility would also enhance the ability of nurses to address these emerging challenges [19]. Continued professional development programs focused on reproductive health advocacy would further equip nurses with the tools they need to influence public health policy and improve access to infertility care [68]. Finally, there is a strong case for integrating nurses more directly into policy-making bodies that shape reproductive health agendas [69]. Nurses possess unique insights from their frontline experiences and can advocate for policies that reflect the needs of patients, particularly in underserved communities [32][69]. By playing a more active role in shaping reproductive health policy, nurses can push for expanded access to infertility prevention services, funding for public health education, and regulatory measures to mitigate environmental factors that contribute to infertility [69]. This advocacy can also promote gender equity in fertility care, ensuring that both male and female infertility issues are addressed in health policies. Integrating nurses into these decision-making bodies will help ensure that policies are informed by evidence-based practices and the practical realities of healthcare delivery [68][69].

## Conclusion

This review highlights the essential role of nurses in infertility prevention and health promotion within public health frameworks. Through interventions such as awareness campaigns, reproductive health counseling, screening programs, and advocacy efforts, nurses are uniquely positioned to address the modifiable risk factors associated with infertility and improve reproductive health outcomes globally. By engaging with communities, nurses help raise awareness about fertility risks, provide critical early detection services, and advocate for policies that increase access to infertility care.

The findings reaffirm that public health nurses, with their holistic approach and community-based presence, are crucial to reducing infertility rates. Their work not only focuses on prevention but also addresses the psychosocial and educational needs of individuals and communities. Moving forward, further research and improvements in nursing education, along with greater involvement in policy-making, will strengthen the role of nurses in mitigating the global burden of infertility.

## Conflict of Interest

None.
